# Supplementary material for: Antibodies with Engineered Fc Domains Having Absolute Binding Selectivity to either FcγRIIa or FcγRI Delineate the Respective Effector Phenotypes by Human Monocytes and Macrophages
Source: bioRxiv. 2026 Sep 4:2026.08.31.748147. Preprint. [Version 1] doi: 10.64898/2026.08.31.748147 (PMC13684468; doi:10.64898/2026.08.31.748147)
Supplement: Supplementary file 1 [file NIHPP2026.08.31.748147v1-supplement-2.pdf]

SI Table 1

bioRxiv preprint doi: https://doi.org/10.64898/2026.08.31.748147; this version posted September 4, 2026. The copyright holder for this preprint (which was not certified by peer review) is the author/funder, who has granted bioRxiv a license to display the preprint in perpetuity. It is made available under a CC-BY 4.0 International license.

Variant	Mutations
Fc2	K246Q, T260A, S298G, T299A, A378N, N390D
$Fc2_{KG}$	G236A, K246Q, T260A, S298G, T299A, A378N, N390D
Fc5	T299L, E382V, M428I
LALAPG	L234A, L235A, P329G

bioRxiv preprint doi: https://doi.org/10.64898/2026.08.31.748147; this version posted September 4, 2026. The copyright holder for this preprint (which was not certified by peer review) is the author/funder, who has granted bioRxiv a license to display the preprint in perpetuity. It is made available under a CC-BY 4.0 International license.

SI Table 2

	Fc2	Fc 2: FcγRIIa$_{R131}$	Fc 2: FcγRIIa$_{H131}$
**Data Collection**			
Space Group	$P2_12_12_1$	$P2_12_12$	$P2_12_12$
Wavelength (Å)	1.0332	0.97741	1.03320
**Cell Dimensions**			
a, b, c (Å)	69.55, 77.55,112.86	99.78,125.35, 140.16	99.96, 126.79, 140.27
α, β, γ (˚)	90.0, 90.0, 90.0	90.0, 90.0, 90.0	90.0, 90.0, 90.0
Resolution (Å)	50.0-2.6 (2.64-2.6)*	50.0-2.7 (2.75-2.70)	50-3.1 (3.15-3.1)
R_{sym} or R_{merge}	0.110 (0.931)	0.143 (0.829)	0.145 (0.599)
I/σI	14.335 (1.474)	10.342 (1.514)	7.325 (1.603)
Completeness (%)	97.5 (98.4)	96.1 (98.4)	90.3 (93.9)
Redundancy	3.4 (3.5)	4.6 (4.7)	3.6 (3.6)
**Refinement Statistics**			
Resolution Range (Å)	47.06 - 2.60	49.64 - 2.70	39.25 - 3.10
No. of Reflections	18878	46863	29732
$R_{work}/R_{free}{}^{\eta}$	0.19 / 0.24	0.22/0.28	0.22/0.28
**No. of Atoms**			
Protein	3327	9534	9465
Water	36	51	0
Sugar	0	70	56
**Bfactor(Å²)**			
Protein	55.5	46.4	54.9
Water	54.1	41.3	N/A
Sugar	N/A	79.6	80.4
**R.m.s. Deviations**			
Bond Lengths (Å)	0.008	0.005	0.003
Bond Angles (deg)	0.932	1.001	0.633
**Ramachandran Plot**			
Favoured(%)	98.8	95.03	95.59
Allowed(%)	1.2	4.97	4.33
Outliers(%)	0.0	0.00	0.08
Molprobity Score	1.64 (99th percentile)	1.97 (98th percentile)	1.60 (100th percentile)
Clash Score	8.22 (99th percentile)	8.71 (99th percentile)	5.49 (100th percentile)

* Statistics in the last shell

$^{\eta}$A test set of 2000 reflections was kept aside during refinement

bioRxiv preprint doi: https://doi.org/10.64898/2026.08.31.748147; this version posted September 4, 2026. The copyright holder for this preprint (which was not certified by peer review) is the author/funder, who has granted bioRxiv a license to display the preprint in perpetuity. It is made available under a CC-BY 4.0 International license.

SI Figure 1

A

K_D (µM) ±SE	FcγRIIIa$_{V158}$	FcγRIIIa$_{F158}$	FcγRIIIb$_{NA2}$
WT	0.113 ± 0.01	0.640 ± 0.051	3.500 ± 0.220
Fc2	17 ± 2.35	NB	NB
Fc2$_{KG}$	NB	NB	NB

B

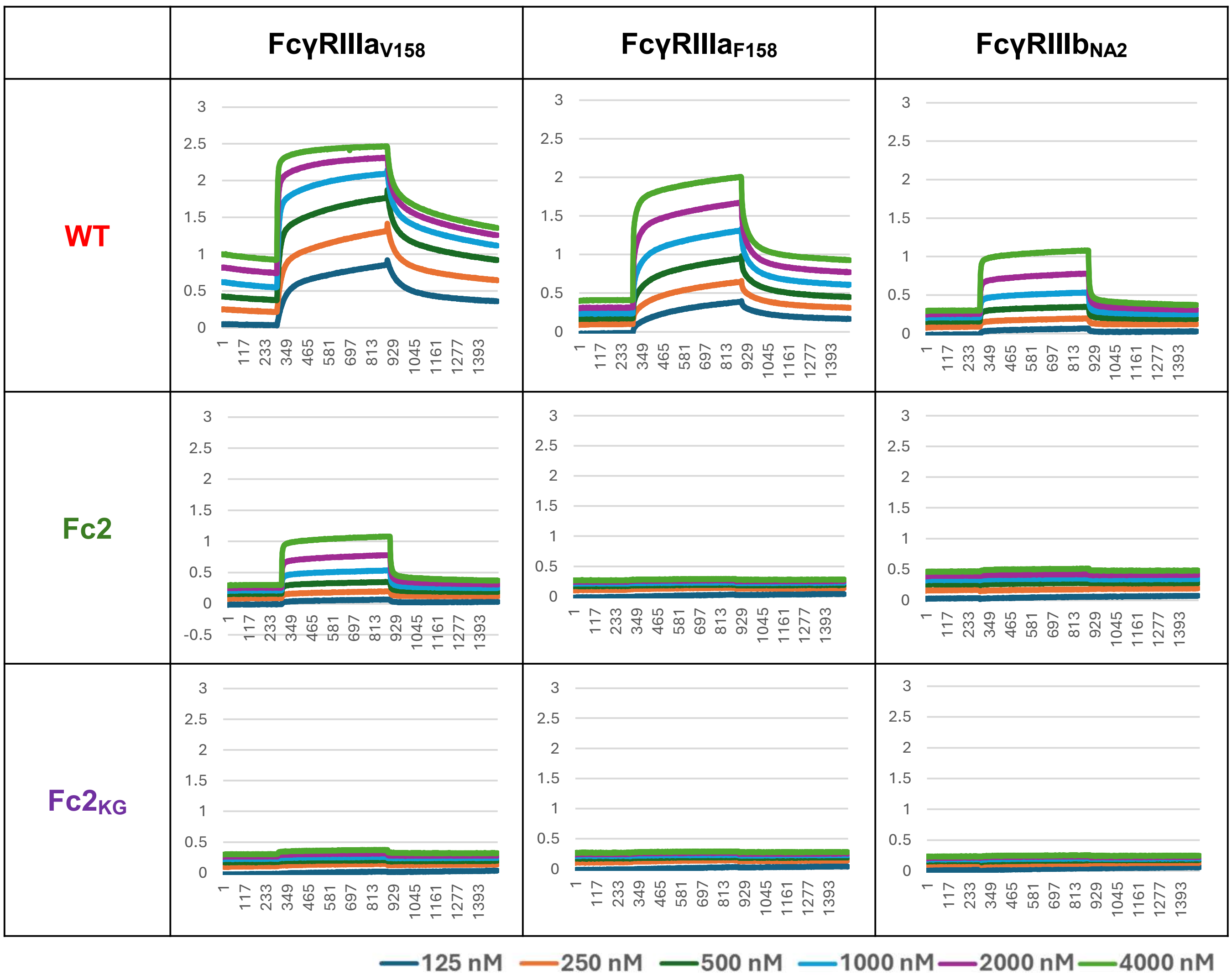

y-axis: response (nm), x-axis: time (s)

bioRxiv preprint doi: https://doi.org/10.64898/2026.08.31.748147; this version posted September 4, 2026. The copyright holder for this preprint (which was not certified by peer review) is the author/funder, who has granted bioRxiv a license to display the preprint in perpetuity. It is made available under a CC-BY 4.0 International license.

SI Figure 2

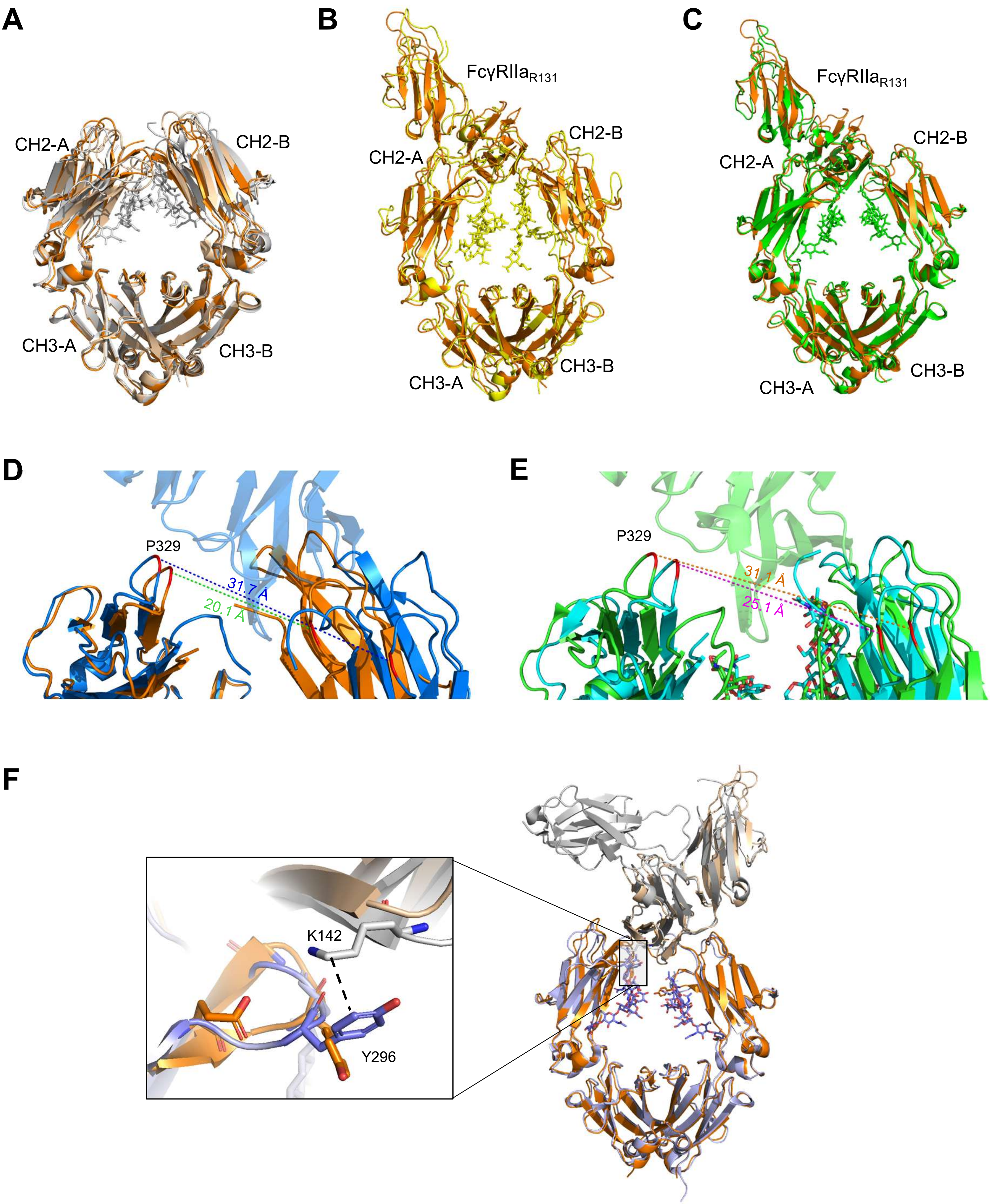

bioRxiv preprint doi: https://doi.org/10.64898/2026.08.31.748147; this version posted September 4, 2026. The copyright holder for this preprint (which was not certified by peer review) is the author/funder, who has granted bioRxiv a license to display the preprint in perpetuity. It is made available under a CC-BY 4.0 International license.

SI Figure 3

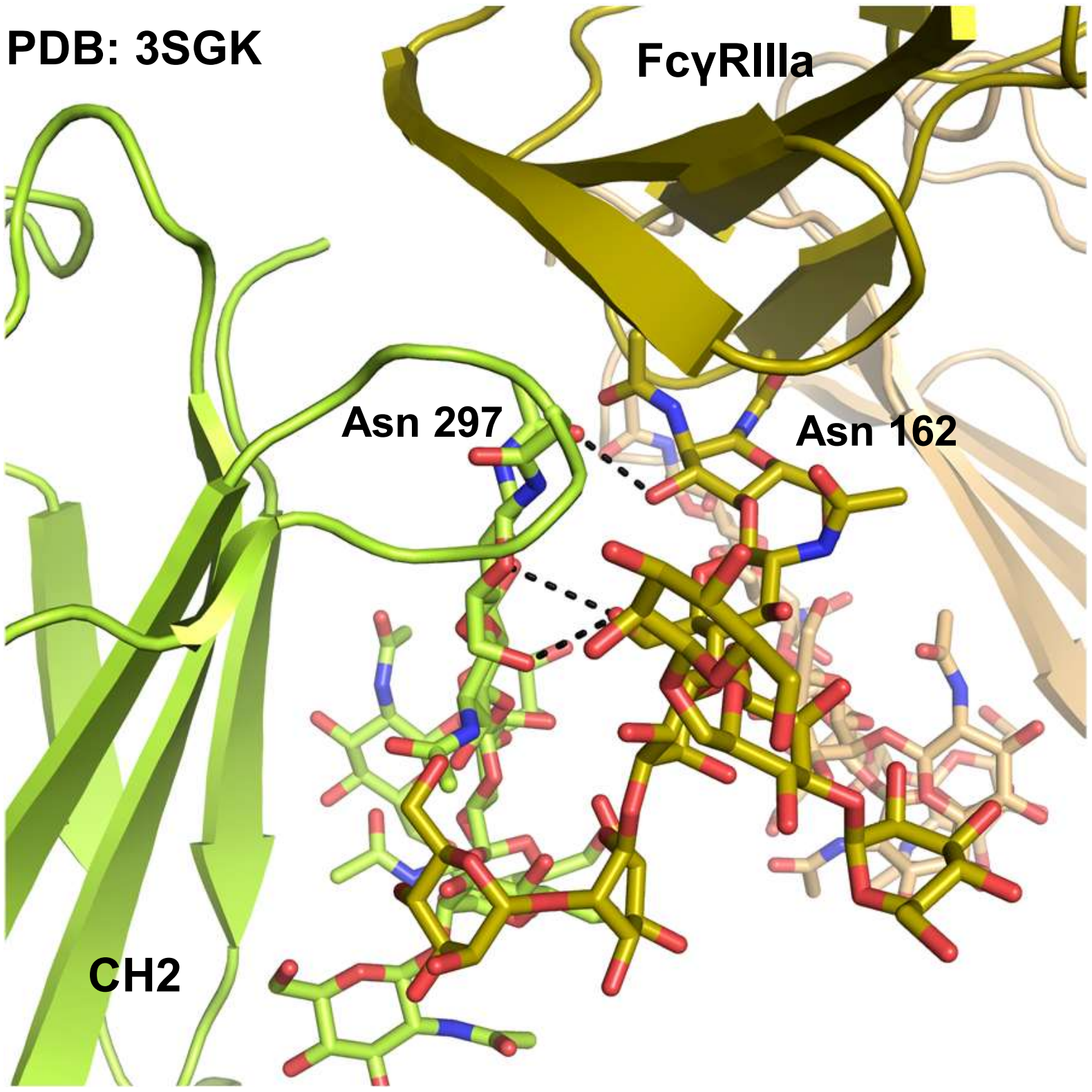

bioRxiv preprint doi: https://doi.org/10.64898/2026.08.31.748147; this version posted September 4, 2026. The copyright holder for this preprint (which was not certified by peer review) is the author/funder, who has granted bioRxiv a license to display the preprint in perpetuity. It is made available under a CC-BY 4.0 International license.

SI Figure 4

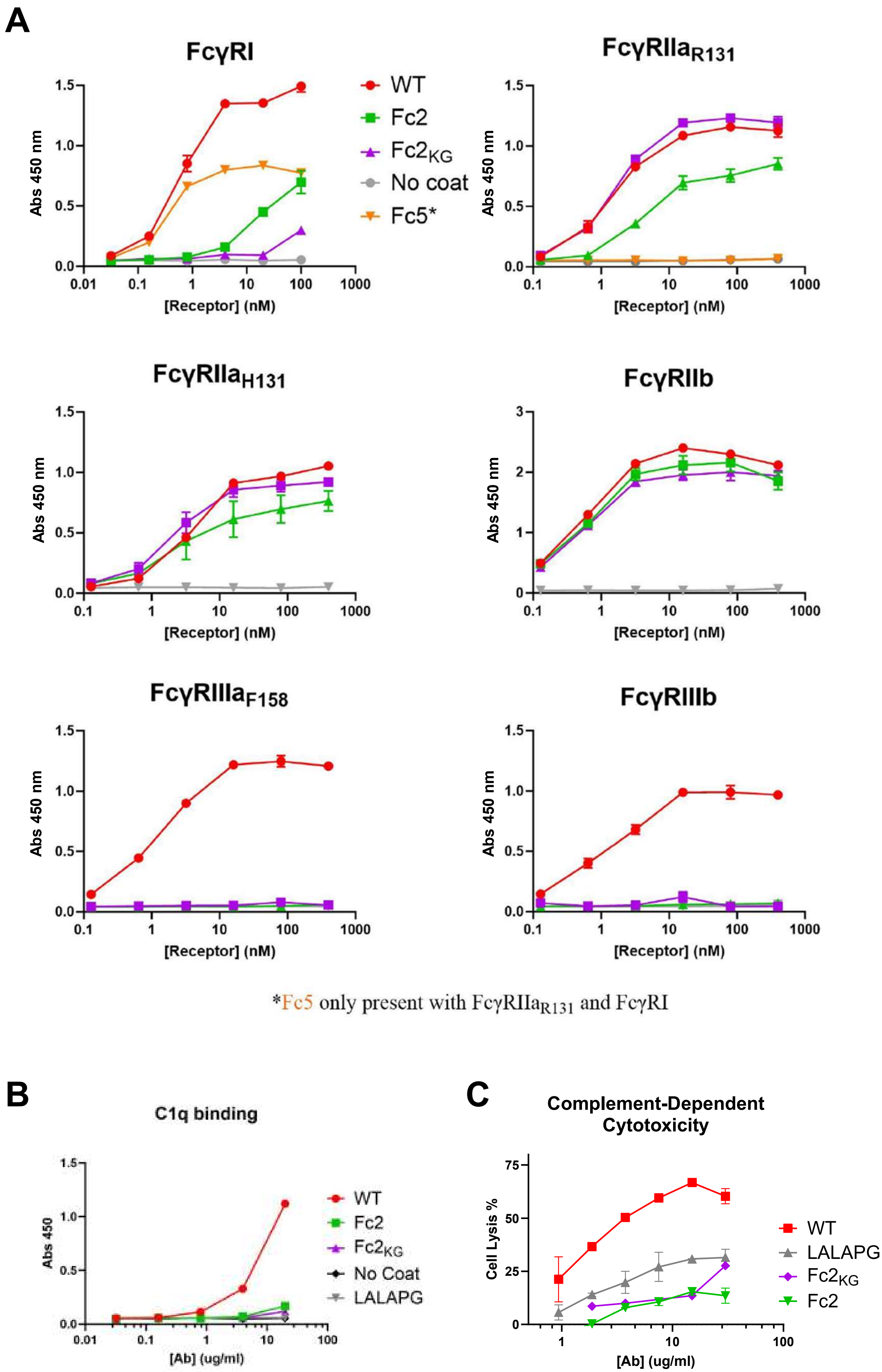

bioRxiv preprint doi: https://doi.org/10.64898/2026.08.31.748147; this version posted September 4, 2026. The copyright holder for this preprint (which was not certified by peer review) is the author/funder, who has granted bioRxiv a license to display the preprint in perpetuity. It is made available under a CC-BY 4.0 International license.

SI Figure 5

A

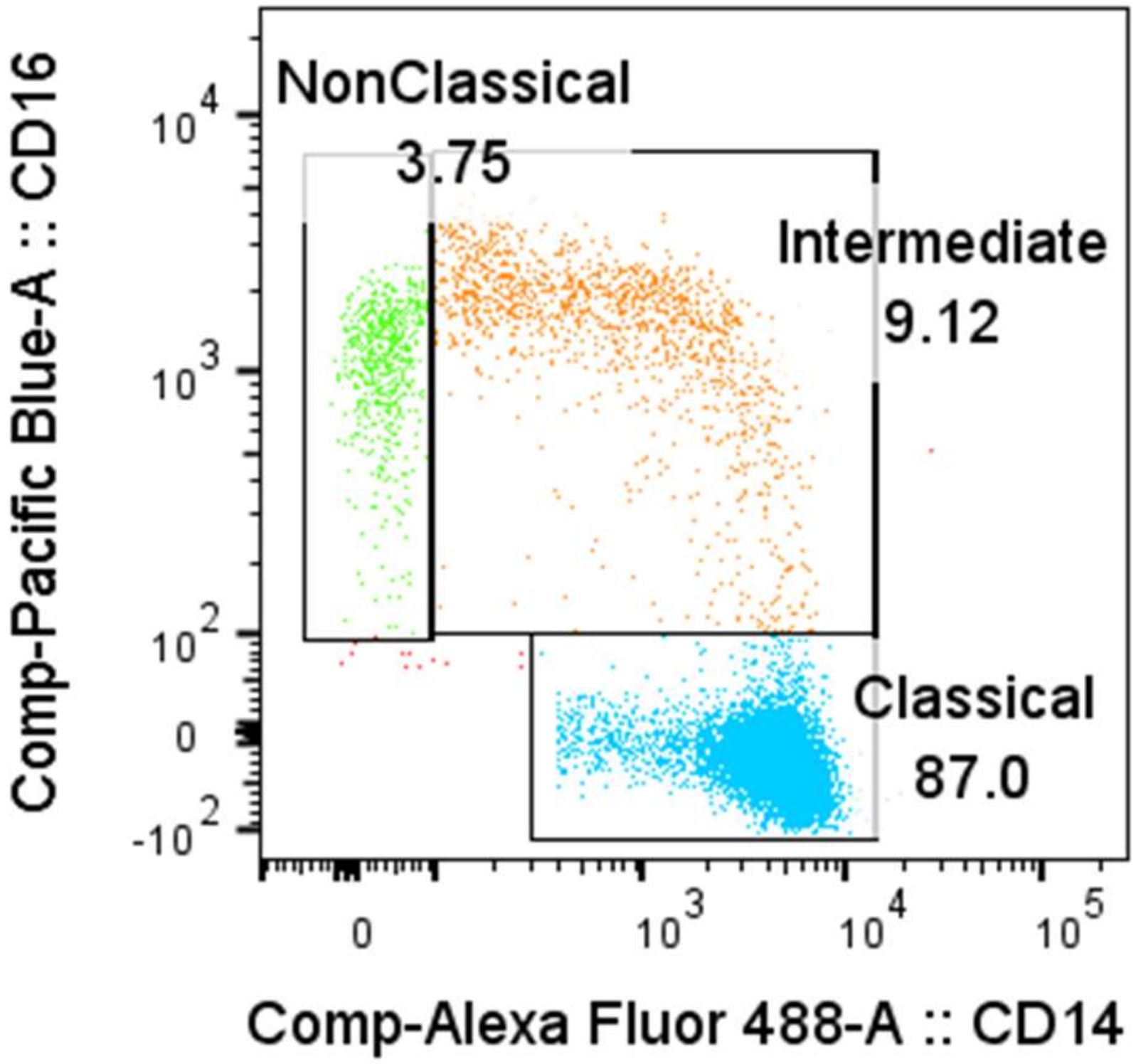

B

Percent Expression				
Cell Type	FcγRI<br>CD64	FcγRIIa<br>CD32a	FcγRIIb<br>CD32b	FcγRIII<br>CD16
Classical	99.8%	99.0%	0.5%	0.0%
Intermediate	70.1%	99.0%	1.2%	100.0%
Nonclassical	1.5%	97.6%	0.0%	100.0%

bioRxiv preprint doi: https://doi.org/10.64898/2026.08.31.748147; this version posted September 4, 2026. The copyright holder for this preprint (which was not certified by peer review) is the author/funder, who has granted bioRxiv a license to display the preprint in perpetuity. It is made available under a CC-BY 4.0 International license.

SI Figure 6

A

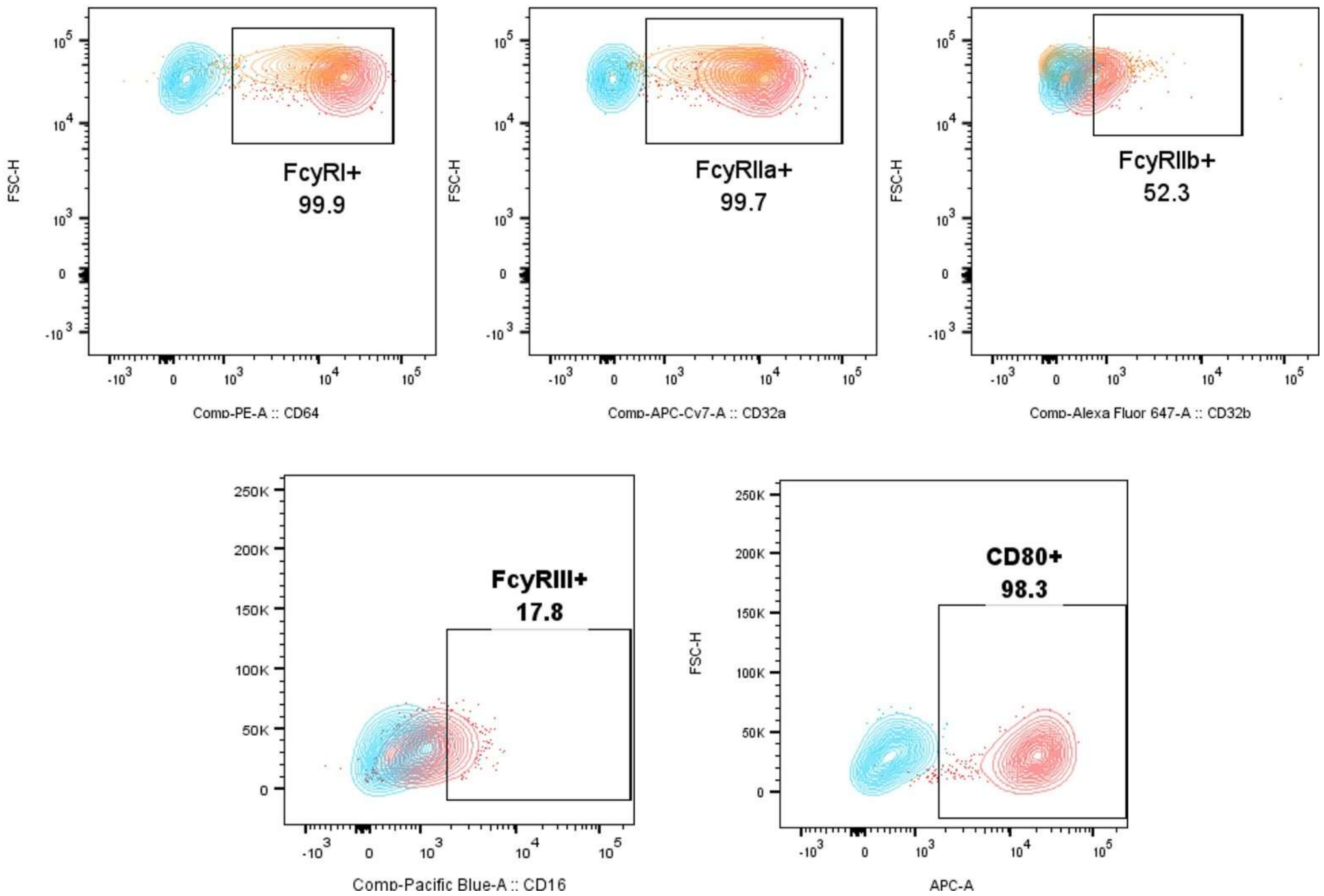

B

	% Positive	
	**Donor A (red)**	**Donor B (orange)**
**FcγRI/ CD64**	99.9	98.0
**FcγRIIa/ CD32a**	99.7	97.5
**FcγRIIb/ CD32b**	52.3	48.1
**FcγRIII/ CD16**	17.8	18.1
