# Supplementary material for: Antibodies with Engineered Fc Domains Having Absolute Binding Selectivity to either FcγRIIa or FcγRI Delineate the Respective Effector Phenotypes by Human Monocytes and Macrophages
Source: bioRxiv. 2026 Sep 4:2026.08.31.748147. Preprint. [Version 1] doi: 10.64898/2026.08.31.748147 (PMC13684468; doi:10.64898/2026.08.31.748147)
Supplement: Supplementary file 2 [file media-1.pdf]

SUPPLEMENTAL INFORMATION

Table S1. Engineered IgG1 Fc variants and corresponding amino acid mutations.

Table S2. Crystallization parameters for apo Fc2 and co-complex structures of Fc2 with FcγRIIa$_{R131}$ and FcγRIIa$_{H131}$.

Figure S1. BLI analysis of engineered IgG1 Fc variants binding to Fc receptors FcγRIIIa$_{V158}$, FcγRIIIa$_{F158}$, and FcγRIIIb$_{NA2}$. A) Summary of K_D values for rituximab-wt, rituximab-Fc2, and rituximab-Fc2$_{KG}$. All values were calculated using steady-state analysis. **B**) Representative BLI sensorgrams of rituximab-wt, rituximab-Fc2, and rituximab-Fc2$_{KG}$ binding to FcγRIIIa$_{V158}$, FcγRIIIa$_{F158}$, and FcγRIIIb$_{NA2}$. Biosensors immobilized with the indicated Fcγ receptors were dipped into serial concentrations of IgG (125–4000 nM) for association, followed by dipping into buffer alone for dissociation. x-axis: time (s); y-axis: response (nm). Colors correspond to IgG concentrations as indicated.

Figure S2. Structural characterization of Fc2 and conformational changes upon FcγRIIa engagement. A) Superposition of wild-type glycosylated IgG1 Fc (gray, PDB: 3AVE), wild-type aglycosylated IgG1 Fc (beige, PDB: 3S7G), and apo Fc2 (orange). **B)** Superposition of wild-type glycosylated IgG1 Fc:FcγRIIa$_{R131}$ (yellow, PDB: 3RY6) and Fc2:FcγRIIa$_{R131}$ (orange). **C)** Superposition of wild-type glycosylated IgG1 Fc:FcγRIIa$_{R131}$ (green, PDB: 9MCY) and Fc2:FcγRIIa$_{R131}$ (orange). **D)** Superposition of apo Fc2 (orange) and Fc2:FcγRIIa$_{R131}$ complex (blue). The Pro329 Cα–Cα distance between the A and B chains increases from 20.1 to 31.7 Å upon receptor binding, reflecting an outward splaying of the two CH2 domains. **E)** Superposition of wild-type glycosylated IgG1 Fc before (cyan, PDB: 3AVE) and after (green, PDB: 9MCY) binding to FcγRIIa$_{R131}$. The distance between the P329 of the A and B chains of the Fc domain is highlighted to illustrate conformational changes upon receptor engagement. **F)** Proposed structural basis for the reduced binding of Fc2 to FcγRI. Close-up view of the Fc2:FcγRIIa$_{R131}$ complex (orange/beige) superimposed on the wild-type IgG1 Fc:FcγRI complex structure (PDB: 4W4O; blue/gray). Zoomed-in box: FcγRI residue K142 and IgG1 Fc residue Y296 observed in the wild-type IgG1 Fc:FcγRI complex (dashed line) and in the Fc2:FcγRIIa$_{R131}$ complex.

Figure S3. Glycan-mediated contacts at the IgG1 Fc–FcγRIIIa interface. Close-up view of the binding interface between wild-type glycosylated IgG1 Fc (green, CH2-A chain) and FcγRIIIa (gold) derived from the crystal structure PDB: 3SGK. The Asn297-linked glycan of the IgG1 Fc (green sticks) and the Asn162-linked glycan of FcγRIIIa (gold sticks) form direct glycan–glycan contacts (dashed lines).

Figure S4. Fc receptor binding and complement activity of engineered Fc variants. A) Immobilized antibodies were tested for binding to serial dilutions of FcγRII- and FcγRIII-tetramers or FcγRI monomer by ELISA. Representative data shown of one out of two independent experiments run in duplicate. **B**) Binding of serially diluted C1q to immobilized rituximab-wt or rituximab-Fc variants was evaluated by ELISA. **C**) Complement-dependent cytotoxicity (CDC) of Ramos cells opsonized with serially diluted rituximab-wt, rituximab-$Fc2_{KG}$, rituximab-Fc5, or rituximab-LALAPG, followed by incubation with 10% pooled human serum as a complement source. Data shown are representative of two independent experiments performed in duplicate at each concentration. Error bars indicate ± SD.

Figure S5. Flow cytometric characterization of FcγR expression on primary human monocytes. A) The expression of FcγRs on classical ($CD14^{++}$ $CD16^{-}$), intermediate ($CD14^{++}$ $CD16^{+}$) and non-classical monocytes ($CD14^{low}$ $CD16^{++}$) was determined by flow cytometry. **B)** FcγRs expression on blood-derived primary monocytes as determined via flow cytometry. The data shown are from one representative of three experiments.

Figure S6. Flow cytometric analysis of FcγR expression on monocyte-derived M1 macrophages. A) Representative contour plots showing FcγR surface expression on monocyte-derived M(LPS+IFNγ) macrophages from two donors after 9 days of in vitro differentiation and polarization. The blue population represents unstained cells from Donor A, the red population represents Donor A stained with the indicated fluorophore-conjugated antibody, and the orange population represents Donor B stained with the indicated fluorophore-conjugated antibody. CD80 expression was included as a marker of M1 polarization. **B)** Quantitative summary of FcγR surface expression (% positive) on M(LPS+IFNγ) macrophages from two independently differentiated donors. Colors correspond to those in panel (A): Donor A (red) and Donor B (orange). Gating was performed as described in Figure S6A.
